# Prevalence and determinants of post-traumatic stress disorder among road traffic accident survivors: a prospective survey at selected hospitals in southern Ethiopia

**DOI:** 10.1186/s12873-020-00348-5

**Published:** 2020-06-26

**Authors:** Asres Bedaso, Gemechu Kediro, Jemal Ebrahim, Firkru Tadesse, Shewangizaw Mekonnen, Negeso Gobena, Ephrem Gebrehana

**Affiliations:** 1 College of Medicine and Health Sciences, Faculty of Health Sciences, School of Nursing, Hawassa University, Hawassa, Ethiopia; 2 Department of public health, University of Technology Sydney, Faculty of Health, Sydney, NSW Australia; 3 Department of Anesthesia, College of Medicine and Health Sciences, Faculty of Medicine, Hawassa University, Hawassa, Ethiopia; 4 Department of Orthopedics, College of Medicine and Health Sciences, Faculty of Medicine, Hawassa University, Hawassa, Ethiopia

**Keywords:** PTSD, Prevalence, Determinants, RTA survivors, Southern Ethiopia, PCL-S

## Abstract

**Background:**

Post-traumatic stress disorder (PTSD) is prevalent among road traffic accident survivors (RTA), yet the psychological welfare of the persons has largely been ignored as health care professionals focus more on managing physical injuries. Many literatures from other parts of the world have addressed the issue of post-traumatic stress disorder among road traffic accident survivors, but such studies are mostly unavailable in sub-Saharan Africa, especially in Ethiopia. Therefore, this study examined the prevalence and determinants of PTSD among RTA survivors attending selected hospitals in southern Ethiopia.

**Methods:**

Institution based cross-sectional study design was employed from April 1/2018-Sep 30/2019. Data were collected from a sample of consecutively selected 423 RTA survivors through an interviewer-administered technique. A pre-tested post-traumatic stress disorder Checklist-Specific version (PCL-S) tool was used to screen PTSD. Data were entered and analysed using SPSS 22 software. A logistic regression model was fitted to identify determinants of PTSD. An adjusted odds ratio (AOR) with a 95% confidence interval was computed to determine the level of significance with a *p*-value of less than 0.05.

**Result:**

A total of 416 participants were included in the study and the response rate was 98.6%. The prevalence of probable PTSD among RTA survivors was 15.4% (64). After adjusting for the effects of potential confounding variables; time since accident (30–90 days) (AOR = 0.33; 95%CI (0.15, 0.73), history of previous road traffic accident (AOR = 2.67; 95%CI (1.23, 5.77), depressive symptoms (AOR = 2.5, 95% CI (1.10, 6.10)) and common mental disorder (AOR = 12.78, 95% CI (5.56, 29.36)) were significant determinants of PTSD.

**Conclusion:**

The prevalence of probable PTSD in the current study was high (15.4%). Time since accident, history of a previous road traffic accident, having depressive symptoms and common mental disorder were significant determinants of PTSD. RTA survivors attending adult Emergency and orthopedic clinics need to be screened for PTSD and get appropriate management.

## Background

Post-traumatic stress disorder (PTSD) is characterized by symptoms like recurrent, involuntary, and intrusive distressing memories of the traumatic event and dissociative reactions and can be diagnosed from four weeks onward [1]. The symptoms are more severe among individuals experiencing traumatic events compared to individuals experiencing non-traumatic events [2].

Globally, over 50 million people experience trauma through road traffic accidents (RTAs) every year [3]. Road traffic injuries and related deaths have turned out to be huge public health problems in the developing world, where 90% of the world’s deaths due to road traffic injuries are estimated to occur [4, 5] . Road traffic accidents (RTA) can have serious and long-lasting consequences for survivors, both in terms of physical and psychological outcomes. Different studies have shown that involvement in RTA may put individuals at increased risk for different psychiatric disorders, including posttraumatic stress disorder (PTSD), depression and anxiety [6–8]. Particularly, PTSD is now a significant public health issue among RTA survivors [9, 10].

The major confront after experiencing trauma is trouble in identifying persons who will shortly develop PTSD [5]. Even if most individuals exhibit PTSD symptoms in the first few weeks after a trauma, more than 50% improve without any intervention within three months [11]. It is argued that by early identification of individuals at risk of PTSD, we can give treatments in the acute post-disaster phase to prevent PTSD [12]. Based on reports from different studies, the acute stress symptoms resolve within a few weeks and a large number of patients display symptoms within 6 to 8 months after injury [13, 14].

Road traffic injury (RTI) is one of the traumatic experiences, which may lead to acute stress and post-traumatic stress disorder [15, 16]. Currently, Road traffic accidents are a growing public health burden, especially in low and middle-income countries and estimated to be the second most important cause of disability-adjusted life years (DALYs) [16]. Mortality due to RTI in Africa is the highest in the world and there is an occurrence of 28.3 deaths per 100,000 populations [4, 17].

In the USA, Morbidity is mainly caused by accidents due to motor vehicles, which is the leading cause of deaths with 3.5 million reported victims annually [18]. In order to reduce this, the government closely monitors motor vehicle accidents (MVA) to prevent additional issues like a psychological problem (PTSD) other than managing the physical injury only [19]. Also, it is necessary to early diagnose the disorder and give primary care for the victims [20]. Morbidities and mortality caused by road traffic accidents are public health problems in low-income countries, especially in Ethiopia. From the year 2009/2010 to 2012/2013, Ethiopia has experienced a high rate of RTA death which is between the ranges of 129–145 per 10,000 vehicles [21].

European countries have reported a different number of incidence of psychiatric morbidity following a road traffic accident. A study conducted in Germany on 179 patients with injury from car accidents, showed that 18.4% of them developed PTSD [22]. Also, a cross-sectional study conducted in Oyo state Nigeria (*n* = 1105) the prevalence of psychiatric morbidity among RTA survivors was 21.9% [23]. Also, the prevalence of PTSD in a study conducted in Taiwan, United Kingdom, South Eastern Nigeria USA, and Kenya following car accident was estimated to be 82.2% [24], 29.1% [25], 26.7% [26], 51% [27], and 13.3% [28] respectively. A longitudinal study conducted in Bahir Dar town, Ethiopia reported an incidence of 46.5% of PTSD among RTA survivors attending the orthopedic unit [29]. Also, another facility-based cross-sectional study conducted in Addis Ababa city revealed a prevalence of 22.8% of PTSD among RTA survivors [30]. A systematic review found that the rates of PTSD among RTA survivors could range from 2 to 50% across studies [31].

Being female, low income, having the previous history of trauma, family and personal history of mental illness, the occurrence of a physical problem following injury, unemployment, presence of co-morbid psychiatric disorder and lack of social support are the major determinants of PTSD [13, 14, 26, 32–34].

PTSD could result in long-term adverse consequences if left untreated, mainly leads to social and functional impairments of RTA survivors which finally result in a poor quality of life [34]. Different studies from other parts of the world have addressed the issue of PTSD but such studies are largely unavailable in sub-Saharan Africa especially in Ethiopia. Therefore, this study examined the prevalence and determinants of PTSD among RTA survivors attending selected hospitals in southern Ethiopia. The finding could help to improve the detection and integrated management of comorbid PTSD at health facilities together with medical management.

## Methods

### Study design and setting

Institution based cross-sectional study design was conducted at the emergency and orthopedic department of selected hospitals in southern Ethiopia. Data were collected from five purposively selected hospitals (from 57 hospitals of all type) in the SNNP region namely; Hawassa University comprehensive specialized hospital (HUCSH), Dilla University referral hospital (DURH), Yirgalem general hospital, Shashemene referral hospital and Wolaita Sodo Christian hospital from April 1/2018–Sep 30/2019. The hospitals were selected based on the total number of RTA cases they managed 6 months ahead of the data collection period.

Hawassa University Comprehensive Specialized Hospital is a teaching hospital for Medical and other Health Sciences students. It is located 275 km south of Addis Ababa and established in 1996 E.C. It offers service at general and specialty levels including internal medicine, pediatrics and child health, general surgery, gynecology and obstetrics, ENT, neurology, neurosurgery and other services. The hospital is giving service for about 18 million people of the southern region of Ethiopia and neighboring areas of Oromia regional state. Currently, it has 350 beds for inpatient admission services.

Yirgalem general hospital is a public hospital located in Yirgalem town, Sidama zone, in the Southern, Nations, Nationalities, and Peoples Region (SNNPR). The hospital is found about 315 km away from the capital city Addis Ababa and inaugurated in 1966. The hospital served about 65,222 patients in 2017. It serves a catchment population of 4.2 million people, mainly from Sidama zone and other surrounding areas. The hospital is giving the service through four main departments namely: medical, surgical, pediatrics, Gynecology/Obstetrics and three special care units (Medical Intensive Care Unit, Neonatal Intensive Care Unit and Surgical recovery Room) and five clinics (Eye, Anti-retroviral Treatment, Dental, TB and MDR-TB clinics).

Dilla University Referral hospital (DURH) found in Dilla town, Gedeo zone, in the Southern, Nations, Nationalities, and Peoples Region (SNNPR) and found 360 KM away from Addis Ababa. Beyond teaching medical and other health science students, it gives inpatient and outpatient services using departments like internal medicine, pediatrics, general surgery, gynecology/obstetrics, ENT, neurology, Psychiatry, and others.

Shashemene referral hospital is established in 1943 as a leprosy control center. The hospital is found in Kuyera town about 238 km south of the capital city Addis Ababa, Ethiopia. Kuyera town is found near Shashemene which is the administrative center for the west Arsi zone in Oromia Region, Ethiopia. It is one of the oldest hospitals in the Oromia region serving approximately 2.1 million people living in the surrounding area. It gives inpatient and outpatient curative services through different departments.

Sodo Christian hospital is located in Sodo town and found 327 km South of Addis Ababa. The hospital is expected to serve around two million people. The total number of beds in the hospital was about 200. The hospital is well known for its orthopedic services having Ethiopian and foreign health professionals and it is one of 9 training sites in Africa for the Pan African Academy of Christian Surgeons.

In addition to providing surgical, medical, obstetric, and other services, all the above-mentioned hospitals are giving psychiatric service at the outpatient level. In addition to outpatient service, Dilla University referral hospital is now providing inpatient psychiatric service.

### Population

All road traffic accident survivors in the southern region were considered as source population. Road traffic accident survivors attending an emergency and orthopedic department of selected public hospitals in the southern region during the study period were the study population. An individual road traffic accident survivor was the study unit.

### Sample size determination and sampling procedure

Institution based cross-sectional study design was conducted at the emergency and orthopedic department of selected hospitals in southern Ethiopia. The sample size was determined by using a single population proportion formula considering different recommended assumptions (Z = standard normal distribution with a confidence interval of 95% (Z = 1.96), d = 0.05 (Absolute precision, or tolerable margin of error), the anticipated population proportion (*p* = 50%), since no similar study conducted previously in Ethiopia, 50% was used to anticipate the proportion of the population of injured patients who experience PTSD). Adding a 10% non-response rate, the final sample size was 423. Then, proportional allocation of sample size was made for the selected five hospitals.

**Table:** 

Name of Hospital	Allocated Sample size	Collected data
Hawassa Univ. Comp. spec. Hospital (HUCSH)	116	109
Shashemene General Hospital	85	85
Wolaita Sodo Christian hospital	97	97
Dilla University referral hospital (DURH)	66	66
Yirgalem zonal hospital	59	59
Total	423	416

### Data collection and measurements

Two trained psychiatry nurses collected the data from each hospital using interviewer-administered technique. Before the actual data collection pre-test was done on 5% of the sample size to see the reliability of the tool. Injured RTA survivors attending emergency and orthopedic units of each selected hospital and who fulfill the inclusion criteria were interviewed consecutively until the sample size is met.

Any road traffic accident survivor whose age is greater than 18 years and injured on admission for not less than one month and who are voluntary to participate in the study were included. Patients who were unable to give proper information (unconscious, severely ill and unable to communicate) and those who have no accompanying relative or informant to consent for the study were excluded.

The data collection instrument contains socio-demographic factors, clinical factors, and post-traumatic stress disorder Checklist (PCL) questionnaire. The post-traumatic stress disorder Checklist (PCL) (civilian version) assesses symptoms related to stressful experiences. The tool is a 17-item self-report measure reflecting DSM V symptoms of post-traumatic stress disorder. A symptom severity score can be obtained by summing the scores from each of the 17 items that have response options ranging from 1 “Not at all” to 5 “Extremely” [35]. Different studies examined showed that the internal consistency of the tool was above 0.75 [36, 37] and Even if the tool was not validated in Ethiopia we have conducted pretest in Adare Hospital and Cronbach alpha of 0.73 was reported. We did factor analysis to see if there is multicollinearity between items within the tool but we did not get any.

The common mental disorder was assessed using SRQ-20 whether; the respondents had experienced symptoms associated with emotional distress within 4 weeks before the interview [38]. SRQ-20 was validated in Ethiopia [39]. Depressive symptoms among RTA survivors were assessed using PHQ-9 with a 3 point severity scale over the last 2 weeks. It has demonstrated acceptable reliability and validated for use in Ethiopia. A PHQ-9 score ≥ 5 was considered as significant for meeting the symptoms of depressive symptoms [40].

To assess the family history of mental illness, respondents were asked: “Do you know a family member who had experienced a mental illness in the past or currently?” and the responses were yes/no. To examine the substance use history, respondents were asked: ‘Have you ever used any substance in the last 3 months?’ and the responses were yes/no. [41] Supervision was held during data collection and each questionnaire was checked for completeness by a supervisor daily. Average monthly income of study participant < 1.25 USD per day (20*1.25*30 = 750 ETB) labelled as under extreme poverty and, < 2 USD per day (20*2*30 = 1200ETB per month) labelled as under poverty [42].

### Variables and analytical strategy

The outcome variable for the current study is Post-traumatic stress disorder (PTSD)**.** The independent variables include; socio-demographic characteristics **(**age, sex, marital status, monthly income, occupation, education level), duration since the accident, Role during the accident, history of a previous road traffic accident, presence of family or friend in the same accident, depressive symptoms, common mental disorder (CMDs), diagnosed co-morbid medical illness (Diabetes Mellitus, Hypertension, Cardiovascular disorder), substance use in the past 3 months and family history of mental illness.

Data were entered and analyzed using SPSS version 20. Logistic regression analysis was done to see the relationship between each variable with the outcome variable. Firstly, each independent variable was entered into the Bivariable logistic regression model to see its association with PTSD. Secondly, independent variables with *p* < 0.2 in the Bivariable logistic regression were included in the multivariable logistic regression model to control confounding variables. During multivariable logistic regression, a *p*-value of less than 0.05 was considered statistically significant, and an adjusted odds ratio (AOR) with 95% CI was calculated to determine the strength of association. Model fitness was checked using the Hosmer and Lemeshow test, and it was found to be 0.75. Multi-collinearity was checked by the variance inflation factor (VIF) and tolerance.

## Result

### Socio-demographic characteristics

A total of 416 participants were included in the study with a response rate of 98.6%, the remaining 7 excluded from the analysis because of the incomplete questionnaire. The median age of the respondents was 30 years. Among the respondents, 192 (46.2%) were between the age range of 18–25 years, 303 (72.8%) were male, 217 (52.2%) were married and 148 (34.9%) attended secondary education. The median monthly income of the participants was 2000 ETB which ranges from 200 to 10,000 ETB (Table 1).

**Table 1:** Socio-demographic characteristics of road traffic accident survivors attending selected hospitals in southern Ethiopia, SNNPR, Ethiopia, 2019 (***n*** **= 416**)

S.no	Variables	Category	Frequency	Percentage
1	Sex	Male	303	72.8%
		Female	113	27.2%
2	Age	18–25	192	46.2%
		26–35	132	31.7%
		36–45	51	12.3%
		> 46	41	9.9%
3	Marital Status	Single and Widowed	199	47.8%
		Married	217	52.2%
4	Educational level	Unable to read and write	60	14.4%
		Primary education	139	33.4%
		Secondary education	145	34.9%
		College and above	72	17.3%
5	Ethnicity	Oromo	156	37.5%
		Wolaita	64	15.4%
		Sidama	78	18.8%
		Amhara	33	7.9%
		Others	85	20.4%
6	Religion	Orthodox	105	25.2%
		Protestant	197	47.4%
		Catholic	1	0.2%
		Muslim	109	26.2%
		Others	4	1%
7	Occupation	Housewife	32	7.7%
		Civil Servant	55	13.2%
		Farmer	81	19.5%
		Merchant	68	16.3%
		Student	94	22.6%
		Private/NGO	48	11.5%
		Others	38	9.15%
8	Monthly Income	< 750 ETB	45	10.8%
		750–1250 ETB	101	24.3%
		> 1250 ETB	270	64.9%

### Clinical and accident-related factors

From the total study participants, about 50 (12%) had depressive symptoms and 115 (27.6%) had a common mental disorder. 104 (25%) had a history of a previous road traffic accident and 171(41.1%) of the RTA survivors were passengers. 15 (3.6%) had diagnosed with a co-morbid medical illness (Table 2).

**Table 2:** Clinical and accident related factors among road traffic accident survivors (RTA) attending selected hospitals in southern Ethiopia, SNNPR, Ethiopia, 2019 (**n = 416**)

S.n**o**	Variables	Category	Frequency	%
1	Time since accident	30–90 days	344	82.7%
		> 90 days	72	17.3%
2	History of Previous road traffic accident	Yes	104	25%
		No	312	75%
3	Role during accident	Driver and assistant	115	26.9%
		Passenger	171	41.1%
		Pedestrian	130	31.3%
4	Presence of family or friend in the same accident	Yes	37	8.9%
		No	379	91.15
5	Depressive symptoms	Yes	50	12%
		No	366	88%
6	CMDs	Yes	115	27.6%
		No	301	72.4%
**7**	Diagnosed Co-morbid Medical Illness	Yes	15	3.6%
		No	401	96.4%
8	Type of Diagnosed Co-morbid Medical Illness	HTN only	9	60%
		CVD only	3	20%
		DM only	2	13.3%
		> 2 of the above	1	6.7%

*Abbreviations*: *HTN* hypertension, *CVD* cardiovascular disorder, *DM* diabetes mellitus, *CMD* common mental disorder

### Substance use of the respondent’s

**Fig. 1:**
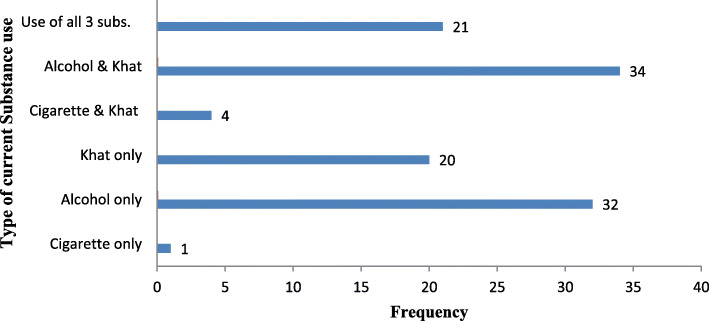
Substance use of road traffic accident survivors attending selected hospitals in southern Ethiopia, SNNPR, Ethiopia, 2019 (**n = 416**)

From the total study participants, 112 (26.9%) use a substance in the past 3 months. Among the total study participants who use a substance, 34 (8.2%) use both alcohol and Khat (Fig. 1).

### The prevalence of PTSD

In the current study, the magnitude of post-traumatic stress disorder was 15.4, 95% CI (12.3–18.8%). The prevalence of PTSD was 15.8 and 14.2% among male and female participants respectively. About 84.4 and 34.4% of participants with PTSD had a co-morbid common mental disorder (CMD) and depression respectively.

### Factors associated with PTSD

After adjusting for confounding variables, multivariable logistic regression analysis revealed, time since accident (30–90 days) (AOR = 0.33; 95%CI (0.15, 0.73), history of a previous road traffic accident (AOR = 2.67; 95%CI (1.23, 5.77), having depression (AOR = 2.5, 95% CI (1.10, 6.10)) and common mental disorder (CMDs) (AOR = 12.78, 95% CI (5.56, 29.36)) were significant determinants of PTSD (Table 3).

**Table 3:** Bivariable and multivariable logistic regression analysis of determinants of PTSD among RTA survivors attending selected hospitals in southern Ethiopia, SNNPR, Ethiopia, 2019 (**n = 416**)

Variable	PTSD		COR (95% CI)	AOR (95%CI)	***P*** value
	No	Yes			
Sex					
Female	97	16	0.87 (0.48, 1.62)		
Male	255	48	RC	RC	
Age					
18–25	165	27	0.68 (0.28,1.62)		
26–35	113	19	0.69 (0.28, 1.73)		
36–45	41	10	1 (0.36, 2.84)		
> 46	33	8	RC	RC	
Marital Status					
Single and Widowed	168	31	1.03 (0.6, 1.75)		
Married	184	33	RC	RC	
Educational level					
Unable to read and write	50	10	1.4 (0.53, 3.71)	0.78 (0.14, 4.31)	0.78
Primary education	121	18	1.04 (0.44, 2.45)	0.75 (0.17, 3.24)	0.69
Secondary education	118	27	1.6 (0.71, 3.61)	1.42 (0.36, 5.63)	0.62
College and above	63	9	RC	RC	
Occupation					
House wife	25	7	RC	RC	
Civil servant	50	5	0.35 (0.1, 1.24)	0.38(.051, 2.83)	0.34
Farmer	65	16	0.87 (0.32, 2.39)	0.91 (0.25, 3.21)	0.91
Merchant	52	16	1.09 (0.4, 3.01)	1.1 (0.28, 4.47)	0.88
Student	87	7	0.28 (0.09, 0.89)	0.37 (0.08, 1.63)	0.19
Private/NGO	40	8	0.71 (0.23, 2.21)	0.36 (0.07, 1.82)	0.22
Others	33	5	0.54 (0.15, 1.9)	0.63 (0.13, 3.1)	0.57
Monthly Income					
< 750 ETB	40	5	0.62 (0.23, 1.67)		
750–1250 ETB	87	14	0.80 (0.42, 1.54)		
> 1250 ETB	225	45	RC	RC	
Time since accident					
30–90 days	311	33	0.14 (0.078, 0.25)	0.33 (0.15, 0.73)	0.006*
> 90 days	41	31	RC	RC	
Role during accident					
Driver and assistant	92	23	RC	RC	
Passenger	147	24	1.66 (0.84, 3.29)	0.72 (0.28, 1.84)	0.49
Pedestrian	113	17	1.08 (0.55, 2.11)	0.63 (0.22, 1.77)	0.38
History of previous road traffic accident					
Yes	62	42	8.9 (4.97, 16.01)	2.67 (1.23, 5.77)	0.013*
No	290	22	RC	RC	
Presence of family or friend in the same accident					
Yes	33	4	0.64 (0.22, 1.88)		
No	319	60	RC	RC	
Depression					
Yes	28	22	6.06 (3.18, 11.54)	2.58 (1.10, 6.10)	0.029*
No	324	42	RC	RC	
CMDs					
Yes	61	54	25.76 (12.42, 53.4)	12.78 (5.56, 29.36)	<0.001*
No	291	10	RC	RC	
Diagnosed Co-morbid Medical Illness					
Yes	13	2	0.84 (0.18,3.82)		
No	339	62	RC	RC	
Substance use in the past 3 month					
Yes	90	22	1.52 (0.86, 2.69)	0.81 (0.35, 1.86)	0.61
No	262	42	RC	RC	
Family history of mental illness					
Yes	6	2	1.86 (0.36, 9.42)		
No	346	62	RC	RC	

*P < 0.05(variables significantly associated with PTSD), *Abbreviation*: *COR* crudes odds ration, *AOR* adjusted odds ration, *CI* confidence interval

## Discussion

Post-traumatic stress disorder (PTSD) is the most common psychopathology and important public health problem among survivors of a road traffic accident (RTA). The finding revealed that a high proportion of RTA survivors who attended the emergency and orthopedic unit of selected hospitals had probable PTSD (15.4, 95% CI; 12.3–18.8). The possible reason for the high burden of PTSD among RTA survivors in the current study might be due to inadequate psychological care in the orthopedic and emergency clinic of each hospital, severity of the trauma, delayed insurance claims and financial shortage for the RTA survivor. The prevalence of the current study was in line with the study done in Kenya 13.3% [28].

However, the prevalence of PTSD in the current study was lower than the study conducted in Germany 18.4% [22], Taiwan 82.2% [24], UK 29.1% [25], South Eastern Nigeria 26.7% [26], USA 51% [27], Iran 19.2% [43], Addis Ababa, Ethiopia 22.8% [30], Bahir Dar town, Ethiopia 46.5% [29] and Serbian study 36% [44]. The reason for the variation might be because of the difference in the socio-economic and geographical nature of the study area, variation in sample size, and difference in utilization of tools used to assess Post-traumatic stress disorder.

The prevalence of PTSD in the current study was higher than the prevalence of PTSD in the general population in the USA (7.8%) [45], Australia (1.1%) [46], Canada (9.2%) [47] and Cape Town, South Africa (12.2%) [32]. The possible reason for the variation might be the difference in the time point of PTSD assessment, which is in South African, the study was conducted 6 months of the post-accident period, but the current study assessed all RTA survivors from 1 month of the post-accident period.

In the current study, time since accident is significant determinants of post-traumatic stress disorder; the odds of developing PTSD among those who experienced RTA 30–90 days before data collection time were 67% (AOR = 0.33; 95% CI (0.15, 0.73) less likely to develop PTSD compared with victims who were injured after 90 days of data collection time. The possible reason might be the decline over the course of the illness as time goes far. This is because the level of stress decreases over time and increased the chance of developing PTSD mainly explained by short duration [48]. This finding was comparable to studies conducted in California [33] and South Africa [32].

Having a history of previous road traffic accidents was also a significant determinant of post-traumatic stress disorder; the odds of developing PTSD among road traffic accident survivors who have the previous history of RTA were 2.6 times (AOR = 2.67; 95% CI (1.23, 5.77) more likely to develop PTSD compared with their counterpart. This could be because having additional trauma could increase the risk of developing PTSD and other psychiatric disorders, which could also lead to deteriorated quality of life. Also, individuals who have experienced a previous road traffic accident, are more susceptible to develop PTSD. The stress of the trauma can have a cumulative effect, and a new traumatic experience can exacerbate the negative effects of previous trauma [49].

A pre-traumatic depression was also reported to be a determinant of PTSD. Those patients with depression were 2.5 times (AOR = 2.5, 95% CI (1.10, 6.10)) more likely to develop PTSD compared with their counterparts. Also, having a pre-traumatic common mental disorder (CMD) was significantly associated with PTSD. Those patients with CMD were more than 12 times (AOR = 12.78, 95% CI (5.56, 29.36)) more likely to develop PTSD compared with those who did not have a common mental disorder. This could be, because of having additional psychiatric disorders could increase the risk of developing PTSD and other mental illness which in turn leads to impaired functional status and decreased quality of life. Also having comorbid mental illness might interfere with a person’s ability to fully process the impact of the traumatic event and their emotions about the event, which will hinder the recovery process. The finding was supported by studies conducted in South Africa, Cape Town [45], and Nigeria [26].

Over all, as it was hypothesized the burden of PTSD was high among RTA survivors attending emergency and orthopedic clinic of selected hospitals in southern region. Also, possible determinants of PTSD were identified which needs consideration while screening for PTSD among RTA survivors.

### Strength and limitations

The current study has numerous strengths. First, selection of study participants from multiple hospitals which addressed wide geographical areas of the region. Second, we used a standardized data collection tool for measuring PTSD (PCL-S). However, the study has some limitations; the tool PCL-S used to assess PTSD was screening rather than a diagnostic tool, the cross-sectional nature of the study (unable to identify a cause-effect relationship), and non-random sampling technique used to select participants are the major ones.

## Conclusion and recommendation

The finding in the current study revealed the prevalence of PTSD was high (15.4%). Time since the accident (30–90 days), history of a previous road traffic accident, pre-trauma depressive symptoms, and common mental disorder (CMDs) were determinants of PTSD among RTA survivors.

We would like to recommend establishing a coordinated triage for prehospital transfer, psychological care in the emergency and orthopedic units, and early screening of PTSD to facilitate appropriate referral to the psychiatry clinic. Also, beyond routine screening of PTSD, ensure the availability of psychological counseling at all the trauma centers and hospitals managing RTA survivors. In long term to reduce the burden of RTA, the regional and federal government needs to set solutions for issues related to road infrastructure, implementing strict traffic rule and law enforcement mechanisms. In future longitudinal studies, have to be conducted to identify other risk factors of PTSD and better participant recruitment strategy needs to be considered.

## Data Availability

All relevant data are included in this article and its supporting document.

## Abbreviations

AOR: Adjusted odds ratio
ASD: Acute stress disorder
CI: Confidence interval
CMD: Common mental disorder
COR: Crudes odds ration
DALYs: Disability adjusted life years
DM: Diabetes Mellitus
DURH: Dilla University Referral Hospital
HUCSH: Hawassa University Comprehensive Specialized Hospital
IRB: Institutional Review Board
MINI: Mini International Neuropsychiatric Interview
OPD: Outpatient Department
PHQ-9: Patient health questionnaire
PTE: Post traumatic event
PTSD: Post traumatic stress disorder
RTA: Road traffic accident
RTI: Road traffic injury
WHO: World Health Organization
